# Rowing Against the Tide: Building Healthier Societies Is Difficult: Comment on "How to Build Healthy Societies: A Thematic Analysis of Relevant Conceptual Frameworks"

**DOI:** 10.34172/ijhpm.9089

**Published:** 2025-08-26

**Authors:** José-Ramón Repullo-Labrador

**Affiliations:** National School of Public Health, Madrid, Spain.

**Keywords:** Public Health, Health in All Policies, Healthy Societies, Health Services Research

## Abstract

The article by Nambiar et al reviews the literature on the influence of policies from outside the health sector, proposing Policy Levers and Enablers to enhance their positive contribution. The regulatory and fiscal lever has a central role, but it should be better articulated with the set of vectors of good governance. Intersectoral action is a complex but necessary axis of work. And it is necessary to change the way of measuring progress and social well-being, but the purpose exceeds the scope and legitimacy of the health sector. In a hostile political environment, it would be advisable to draw up more defensive strategies to defend achievements and avoid setbacks.

## Introduction: The Construction of Healthier Societies

 In the article we are discussing, Nambiar et al^1^ make an extensive and brilliant review of 68 documents selected since 1974 that refer to actions outside the health sector that influence health. After the COVID-19 pandemic, the topic of building healthy societies intensified when the relevance of the effects of the social determinants of health was observed in the impact of the pandemic, particularly the inequality in the treatment of disadvantaged social groups. This context has generated a renewed impetus to review and rethink public interventions and policies, so that they generate an environment more conducive to improving health.

 A first comment would qualify the concept of “healthy societies” by “healthier societies”: there are gradients in how healthy societies can be, which are also complex, multidimensional and dynamic.

 The data allow for various interpretations: in the 21st century, global life expectancy increased by more than 6 years (from 66.8 in 2000 to 73.1 in 2019); Life expectancy in good health has also grown, although somewhat less (from 58.1 to 63.5). Has this improvement been a success of the health sector, or an effect of improvements in the well-being of the population and public policies in other sectors?^2^

 In addition, health objectives are only one part of well-being: When individuals and societies assume behaviors that involve health risks, there is a “trade off” between benefits and risks; Smoking, playing extreme sports, or making a vacation trip involves taking risks. Public health is interventionist by nature, but it must accept that the autonomy of the will of citizens ends up determining the degree to which they incur in activities or consumption that reduce their opportunities for health and well-being.

 At the level of societies, it also happens: Putting the economy ahead of health is a daily reality, which is exacerbated in times of financial or fiscal crisis. Curiously, the pandemic showed that a health problem can stop the economy (health as a precondition for economic development); It does not seem that this hard lesson has been assimilated too much by political decision-makers.

## Levers to Build Healthier Societies

 The aforementioned work seeks to scrutinize the instrumental elements (“how”) that have been used to facilitate the connection between the policies that are developed in the health sector, and those that can contribute from other sectors to generate healthier societies. To this end, both the “levers” that can be used for this purpose and the facilitating elements that condition the operation of these levers are reviewed. In an ingenious cube, the relationship between policy levers, enablers and levels of application (global, national, and local) is drawn.

 The three levers are certainly important, although their nature and scope are quite different.

 The first refers to the *regulatory and fiscal sphere*. Using this lever requires that the public authority has institutional power, the will to implement policies based upon the general interest, and the competence to design and conduct processes and their implementation. The principles of Good Governance formulated in the acronym TAPIC (Transparency, Accountability, Participation, Integrity and Political Capacity) are very applicable.^3^ When states are weak (or failed) it is unlikely that this lever can be activated; even in states with stronger institutions, but with minority governments or weak parliamentary support, its implementation can be affected by the influence of well-resourced interest groups that have learned to selectively influence policy agendas and content.

 The second lever speaks to us of *inter-sectoral action*, and of the difficulty of crossing the barriers between sectors. The preaching of incorporating a health vision into all policies is reasonable and well-intentioned, but it seems not very effective. The authors point out the bias towards the technical dimension in intersectoral initiatives, to the detriment of the analysis of the power dynamics that exist and are triggered by the proposed changes. For example, on this website of the European Observatory of Health Systems and Policies, the contents of a large number of health policies can be traced, with the technical contents dominating over the contextual conditions and the management of power relations.^4^

 It should be noted that there is a deeper problem at work: each sector has its own worldview, specific authorities and stakeholders; the barriers between “ministries” are much stronger than imagined. Getting agriculture and livestock authorities to embrace a health approach requires more than just persuasion. Even in the relationship of close sectors, such as health and social services, there is usually a zero-sum game: what benefits one can be a burden for the other (for example, early discharges from hospitals can overload home social services or help for families).

 The third lever is somewhat more abstract: *to redefine the way we conceive, and measure social progress* is to enter a real battle of values. In the utilitarian postmodernity of the 21st century, it is rowing against the tide: The more profit and individualism are enthroned, the less opportunity there is to displace gross domestic product and short-sighted measures of profit by other metrics of well-being and happiness. It is, however, an essential ideological struggle, although its natural terrain is generalist political debate. When we get into health policies we have a dilemma: Do we use utilitarian arguments of efficiency, or does this incorporate us into the questionable dominant narrative and reinforce it? Do we use the economic gain for society from an anti-smoking policy or coverage of undocumented immigrants?... Does not doing so mean devaluing the arguments of its benefits in health and equity? And what to do with what is not cost-effective... and yet it is socially and healthily necessary?

## Enablers to Apply the Policy Levers

 The enablers pointed out by the authors are closely related to the levers.

*Political Will and Accountability* tells us about the degree of institutional maturity of political power, and how to stimulate it by identifying and reducing the capture of public decision-makers by interested agents; it may be good ideas to give transparency to the problems in parliaments or to seek a disaggregated approach where the effects (costs and benefits) of its application on citizens and social groups are better identified.

 Community *mobilization and action* is essential: It imposes costs and restrictions on the action of influential stakeholders that influence policies behind the scenes and can serve to slow down or stop processes that are very harmful to the health of the population, or also to defend necessary changes that have powerful actors that play against them. But we must bear in mind that pressure groups learn quickly and have resources to influence third parties: For example, the infiltration of patient associations by the technology and pharmaceutical industry, which allows them to activate their protests to accelerate or unblock the authorization of innovations for which the authorities have reasonable doubts about their efficiency.^5^

 The importance of *public education* is also raised, so that well-educated and informed citizens are able to understand and support the interventions that are proposed based on scientific evidence. Here too there are unfavorable changes in political and social culture: The expansion of hoaxes, the influence of magical-mythical interpretations, and the penetration of pseudoscience and pseudo therapies form a tapestry of irrationality that has come to infect political power in large countries and that threatens to counteract the efforts of the educational community.

 The third enabler element is the *generation and use of knowledge*. It is a very important field for defending policies that help to build healthy societies; The battle is going to be unequal and bloody: Just look at how political power in some countries is buying theories without scientific foundation, adhering to hoaxes and conspiracies, and defending irrational and extravagant alternatives. The manipulation of data and information becomes a powerful instrument of influence.^6^

 In addition, the profit motive and trickery invade the world of research; With a tide of junk publications and predatory magazines, it is difficult to separate the wheat from the chaff; Access to large real-life databases through observational designs can jeopardize the validity of scientific research: Any mercenary study can look for spurious causal associations to sell an idea or a product, and present it as causality (“scientific evidence”).

## Technocratic or Power Approach: How Far to Analyze?

 The authors criticize the excessive technocratic approach, when initiatives are proposed to build healthier societies through intersectoral action (for example, “health in all policies”), defending a more in-depth analysis, which evaluates the dimension of power and allows better management of the determinants of the behaviors of the different actors.

 But the problem with delving into these determinants is that they end up taking us out of the plane of sectoral analysis, to enter into debates about Politics with capital letters. And this creates a dilemma: Do we leave the plane of analysis of health policies and lose context and perhaps legitimacy? Or do we retreat to a more specific, and therefore self-limiting and technocratic realm?

 Nothing especially new: Already Virchow stumbled upon this dilemma when he studied the health problems of Silesian miners and concluded with the famous phrase “medicine is a social science and politics nothing but medicine on a grand scale.”^7^

 But in the postmodern times in which we live, we should revisit this contradiction inherent in political and social analyses that are developed from the concern for the health and well-being of citizens. Can we ignore in the analysis the corrosive leaderships of Netanyahu, Milei or Trump? Climate change, the decline of human rights, the fierce utilitarianism of nations, and the erosion of empathy and solidarity with others is the hostile warp on which we must weave healthy policy initiatives: And it does not seem that we have ahead of us times of strategies of progress, but rather of defensive actions.

 Designing specific actions from the healthcare sector to counteract the effects of social determinants that negatively affect the health of the population is complex, especially if it is not feasible to go beyond the sectoral framework. However, we cannot fail to incorporate upstream interventions targeting other sectors into the vision and mission of healthcare systems. As an example, we can cite an interesting project proposed by the Kings Fund in 2024 and published under the title “Tackling health inequalities: seven priorities for the NHS.” It defines four interconnected pillars to guide actions: (*a*) the wider determinants of health; (*b*) healthy behaviours and lifestyles; (*c*) the places and communities we live in; and (*d*) the support of an integrated health system.^8^

 On this basis, seven areas are identified:

1. Develop a cross-government health inequalities strategy for the 10-year health plan to feed into.
2. Reorientate the National Health Service [NHS] to focus on prevention.
3. Radically change the relationships the NHS has with people and communities, from “power over” to “power with.”
4. Tackle racism and discrimination in the NHS and cultivate a culture of compassion.
5. Enable staff to identify and act on health inequalities and capture learning.
6. Empower place-based partnerships to take more decisions about how NHS money is spent.
7. Actively support local voluntary, community and social enterprise organizations through changes in financial planning and commissioning.

## Hard Times and New Challenges to Prevent Societies From Becoming Less Healthy

 We have a difficult exercise of incursion and containment ahead of us: Learning to go locally-globalizing (from the local or sectoral level connecting with general policy and then applying again into healthy policies).

 Improvements in the analysis of the power dynamics underlying actors’ behaviour can help modulate and redesign levers and enablers; the authors are right in this argument. But these in-vitro analyses by social and political analysts must overcome the barrier of implementation, leaving ample room for real decision-makers to interact with reality and add the wisdom offered by practical experience and iteration, in terms of feasibility, outcomes, and adverse consequences.

 Going deeper into the brilliant phrase of the authors, anti-politics not only does not create healthier societies, but ensures the destruction of the instruments and institutions that allow protecting and promoting the health of the population. This is one of the essential challenges of these times: To defend ourselves from the erosion of the institutional framework and to stop or mitigate the deleterious effect of policies that go against the health of our fellow citizens, especially the poorest and most disadvantaged. It is not an optimistic outlook, and it may seem like a somewhat defensive strategy, but it seems to be what will have to be done, at least in the next 5-10 years.

## Ethical issues

 Not applicable.

## Conflicts of interest

 Author declares that he has no conflicts of interest.
